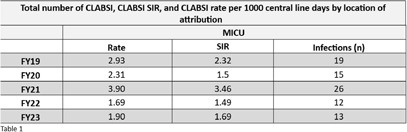# Sustaining the Stewardship – Maintaining CLABSI Prevention Efforts Over the Long Run

**DOI:** 10.1017/ash.2025.291

**Published:** 2025-09-24

**Authors:** Kate Schultz, Hitesh Patel, Kenton Dover, Lauren DiBiase, Lisa Stancill, Fanning Alan, Young Kimberly, Honey Jones, Erin DeMarco, Melody Padgett, Beth Heilman

**Affiliations:** 1 UNC Health Care; 2 UNC School of Medicine; 3 UNC Health; 4 UNC Health Care; 5 UNC Hospitals; 6 UNC Hospitals; 7 UNC Health; 8 UNC Hospitals; 9 University of North Carolina Medical Center - Chapel Hill; 10 University of North Carolina Healthcare; Marsha Taylor

## Abstract

**Background:** Central line-associated bloodstream infections (CLABSIs) are preventable infections associated with poor outcomes. Nationally, the CLABSI standardized infection ratio (SIR) decreased from 2018 to 2019, but those positive results were derailed by the COVID-19 pandemic. From FY21 to FY22, the CLABSI SIR in our facility’s medical intensive care unit (MICU) more than doubled. In March 2021, we created a multidisciplinary central venous access device (CVAD) rounding team to decrease CLABSI in the MICU. **Method:** We conducted a prospective pre-post quality improvement study in an academic, quaternary care hospital with a 30-bed MICU. The decision to implement a multidisciplinary CVAD rounding process was based on a review of published best practices. The study was approved by the UNC Institutional Review Board.

Our team included MICU clinicians, registered nurses, an infection preventionist, a vascular access registered nurse and a “CVAD Liaison”. The CVAD liaison role is a registered nurse trained maintaining aseptic technique during CVAD insertions and educating staff on CVAD maintenance. Each teammate had assigned responsibilities (Fig. 1).

The team rounded weekly on every MICU patient with a CVAD. Components of the rounding process were compliance audits of all CVADs; evaluation of line necessity; targeted education and process improvement. **Result:** This study evaluated the intervention’s impact on CLABSI, CVAD utilization, and maintenance bundle compliance rates. Data were collected for five fiscal years (FY19 to FY23). Following the intervention, the MICU experienced a 57% decrease in CLABSI rates between FY21 and FY22. This reduction was meaningful for patient care, although not statistically significant. Infection rates rose slightly in FY23 but remained lower than from FY19-21 (Table 1). There was no statistically significant difference in the CVAD utilization rate between FY21 and FY22. There was an improvement in the percentage of intact dressings (Fig.2). **Conclusion:** Following the implementation of a multidisciplinary CVAD rounding team, there was a 57% decrease in the MICU’s FY22 CLABSI infection rate from the prior fiscal year. This decrease in CLABSI was sustained with a similar CLABSI rate in FY23. One potential explanation for the CLABSI reduction was increased awareness of the importance of maintaining CVAD dressings (Fig. 2). The primary challenge for this team has been sustaining staff availability. Staff shortages and burnout have made it challenging to find coverage for rounds. Our team began addressing staffing challenges by expanding the pool of interested staff. Sustainment of rounding and CLABSI reduction will require continued monitoring and recruitment.

**Figure:**
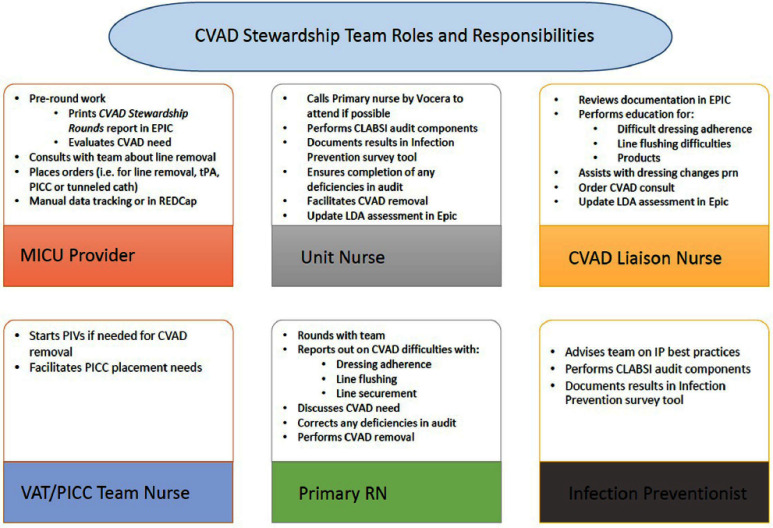

**Figure:**
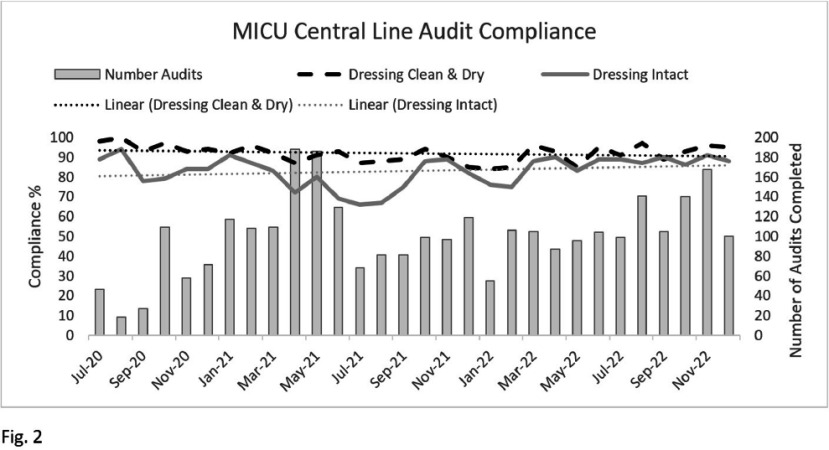

**Figure:**